# The Intricate Relationship Between Pulmonary Fibrosis and Thrombotic Pathology: A Narrative Review

**DOI:** 10.3390/cells13242099

**Published:** 2024-12-18

**Authors:** Giovanni Cenerini, Davide Chimera, Marta Pagnini, Erica Bazzan, Maria Conti, Graziella Turato, Alessandro Celi, Tommaso Neri

**Affiliations:** 1 UO Pneumologia, Azienda Ospedaliero-Universitaria Pisana, 56124 Pisa, Italy; g.cenerini@studenti.unipi.it (G.C.); d.chime91@gmail.com (D.C.); 2 Centro Dipartimentale di Biologia Cellulare Cardiorespiratoria, Dipartimento di Patologia Chirurgica, Medica, Molecolare e dell’Area Critica, Università degli Studi di Pisa, 56124 Pisa, Italy; marta.pagnini@med.unipi.it (M.P.); tommaso.neri@unipi.it (T.N.); 3 Department of Cardiac, Thoracic, Vascular Sciences and Public Health, University of Padova and Padova City Hospital, 35128 Padova, Italy; erica.bazzan@unipd.it (E.B.); maria.conti.2@phd.unipd.it (M.C.); graziella.turato@unipd.it (G.T.); 4 Centro Cardiologico Monzino IRCCS, 20138 Milan, Italy

**Keywords:** idiopathic pulmonary fibrosis, thrombosis, coagulation, anticoagulants, mortality, extracellular vesicles

## Abstract

Idiopathic pulmonary fibrosis (IPF) is associated with a significantly increased risk of thrombotic events and mortality. This review explores the complex bidirectional relationship between pulmonary fibrosis and thrombosis, discussing epidemiological evidence, pathogenetic mechanisms, and therapeutic implications, with a particular focus on the emerging role of extracellular vesicles (EVs) as crucial mediators linking fibrosis and coagulation. Coagulation factors directly promote fibrosis, while fibrosis itself activates thrombotic pathways. Retrospective studies suggest the benefits of anticoagulants in IPF, but prospective trials have faced challenges. Novel anticoagulants, profibrinolytic therapies, and agents targeting protease-activated receptors (PARs) show promise in preclinical studies and early clinical trials. EVs have emerged as key players in the pathogenesis of interstitial lung diseases (ILDs), serving as vehicles for intercellular communication and contributing to both fibrosis and coagulation. EV-based approaches, such as EV modulation, engineered EVs as drug delivery vehicles, and mesenchymal stem cell-derived EVs, represent promising therapeutic strategies. Ongoing research should focus on optimizing risk–benefit profiles, identifying predictive biomarkers, evaluating combination strategies targeting thrombotic, fibrotic, and inflammatory pathways, and advancing the understanding of EVs in ILDs to develop targeted interventions.

## 1. Introduction

Idiopathic pulmonary fibrosis is a severe interstitial lung disease characterized by progressive scarring of the lungs, leading to respiratory failure and death [1]. Despite recent advances in antifibrotic therapies, IPF carries a poor prognosis, with median survival of 3–5 years from diagnosis [2]. Compelling evidence suggests that thrombotic events, including venous thromboembolism (VTE) and acute coronary syndromes (ACS), are more frequent in IPF patients [3,4,5]. The relationship between pulmonary fibrosis and thrombosis appears to be bidirectional. Coagulation factors, particularly thrombin and factor Xa, directly stimulate fibroblast activation and collagen deposition, promoting fibrosis. Conversely, the fibrotic lung environment activates platelets, impairs fibrinolysis, and induces a systemic hypercoagulable state. This self-perpetuating cycle likely accelerates disease progression and increases the risk of fatal thrombotic events. In this review, we comprehensively examine the epidemiological, mechanistic, and therapeutic aspects of the link between pulmonary fibrosis and thrombosis, with a specific emphasis on the emerging role of extracellular vesicles as crucial mediators connecting these two pathological processes. We discuss the challenges and opportunities in targeting thrombotic pathways and leveraging EV-based strategies to improve outcomes in IPF and outline future research directions to advance this field.

## 2. Epidemiological Evidence Linking IPF and Thrombosis

Numerous epidemiological studies have documented an increased risk of venous and arterial thrombotic events in patients with IPF compared to matched controls. A population-based study showed an increased risk for acute coronary syndrome (RR 3.14, 95% CI 2.02–4.87) and deep vein thrombosis (RR 3.39, 95% CI 1.57–7.28) in patients with IPF compared to controls [3]. Similar findings were reported by Sprunger et al. in a large US insurance claims database [6]. Patients with IPF and post-inflammatory pulmonary fibrosis had a significantly higher incidence of pulmonary embolism (HR 1.74, 95% CI 1.42–2.12), deep vein thrombosis (HR 1.41, 95% CI 1.21–1.64), and acute coronary syndrome, (HR 1.37, 95% CI 1.11–1.68) compared to matched controls. The risk of thrombotic events was highest in the first year after diagnosis and remained elevated throughout follow-up. Finally, in a study using data from national Danish registries, Sode and coll. observed an association between an ever-diagnosed episode of venous thromboembolism, considered as a proxy for thrombophilic state, and IPF. The relationship between a thrombophilic state and the development of IPF is corroborated by data from a population-based case–control study that investigated the association between several prothrombotic laboratory abnormality tests and a diagnosis of IPF. The authors demonstrated an odd ratio for IPF of 4.38 (95% CI 2.85 to 6.74) in the presence of any abnormality; the association was particularly strong with elevated FVIII levels, antithrombin deficiency, and increased D-dimer. For the latter, a concentration-dependent effect was also observed. The presence of any prothrombotic state was also associated with a decrease in overall survival [7]. An independent study demonstrated a significantly increased risk of the development of an acute exacerbation of IPF within three months in patients with D-dimer levels ≥ 0.4 ng/mL [8]. The association between IPF and thrombosis is further supported by autopsy studies. In a series of 52 consecutive patients with an acute exacerbation of IPF who underwent autopsy, 9 (17%) had evidence of pulmonary thromboembolism [9]. This is compatible with the hypothesis that thrombotic events could represent a significant contributor to mortality. A meta-analysis has shown that the risk of VTE in IPF patients is approximately two-fold higher than in the general population, with a pooled risk ratio of 2.11 (95% CI: 1.28–3.48) [4].

## 3. Pathogenetic Mechanisms Linking Fibrosis and Thrombosis

**Figure 1:**
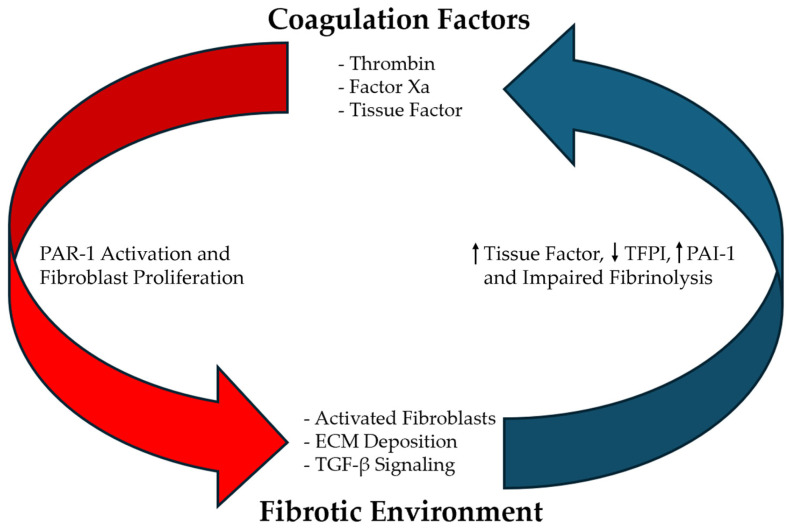
The self-perpetuating cycle between coagulation and fibrosis in idiopathic pulmonary fibrosis. This schematic illustrates the bidirectional relationship between coagulation and fibrosis described in our review. Coagulation factors (thrombin, factor Xa, and tissue factor) activate PAR-1 on fibroblasts, promoting their proliferation. This creates a fibrotic environment characterized by activated fibroblasts, increased extracellular matrix (ECM) deposition, and enhanced TGF-β signaling. The fibrotic environment, in turn, maintains a procoagulant state through increased tissue factor expression, decreased tissue factor pathway inhibitor (TFPI), elevated plasminogen activator inhibitor-1 (PAI-1), and impaired fibrinolysis. This self-reinforcing cycle helps explain the progressive nature of pulmonary fibrosis and its association with thrombotic events.

The epidemiological associations between IPF and thrombosis are supported by data elucidating the pathogenetic mechanisms that link these two processes. The relationship appears to be bidirectional, with coagulation factors promoting fibrosis and the fibrotic environment activating thrombotic pathways (Figure 1).

### 3.1. Direct Profibrotic Effects of the Coagulation Factors

Some coagulation factors (F) function as proteases; their best-recognized role in the coagulation cascade is to cleave proenzymes downstream in the cascade, thereby activating them and making them suitable to act as proteases in the following step. However, besides this long-recognized activity, the same molecules have been shown to cleave protease-activated receptors expressed on numerous cells, including platelets and fibroblasts [10]. PAR are integral membrane proteins that contain both a receptor and its agonist. In the resting conformation, the N-terminal portion of the molecule masks the tethered agonist sequence, making it inactive. Upon cleavage of the N-terminal region through the action of specific proteases, the agonist becomes free to interact with the receptor in an autocrine fashion, thereby initiating downstream signaling pathways. PAR-mediated signaling has been linked to numerous physiologic and pathophysiologic processes, including blood coagulation, inflammation, pain, and wound healing [11]. In vitro studies have shown that thrombin (also referred to as FIIa) induces fibroblast proliferation [12] and procollagen production [13] in a PAR-1-dependent. PAR-1 activation induced by thrombin also promotes myofibroblast differentiation. Myofibroblasts are known to contribute to the increase in extracellular matrix deposition and contractility of lung parenchyma associated with pulmonary fibrosis [14]. Factor Xa, another key coagulation protease, has also been implicated in fibrosis. Like thrombin, factor Xa can activate PAR-1 and stimulate fibroblast activation and differentiation [15]. In addition, factor Xa can promote fibrosis through PAR-1-independent mechanisms, such as the induction of pro-inflammatory and pro-fibrotic cytokines [16]. Animal models of pulmonary fibrosis corroborate these in vitro observations. In PAR-1 deficient mice, total collagen accumulation was significantly reduced upon exposure to bleomycin compared to wild-type controls. Furthermore, pharmacologic inhibition of PAR-1 with the pepducin, P1pal-l2, protects mice from the development of bleomycin-induced fibrosis [17].

### 3.2. Activation of Thrombotic Pathways in the Fibrotic Lung

While coagulation factors promote fibrosis, the fibrotic lung environment itself activates thrombotic pathways, creating a vicious cycle that may contribute to the increased risk of thrombotic events in IPF patients. The alveolar compartment in IPF is characterized by an imbalance between procoagulant and anticoagulant factors, favoring a local hypercoagulable state [18]. BALF from IPF patients contains increased levels of tissue factor (TF), the primary initiator of the extrinsic coagulation cascade, as well as decreased levels of tissue factor pathway inhibitor (TFPI), the endogenous inhibitor of this pathway [19,20]. This shift in the TF/TFPI balance promotes activation of the coagulation cascade and local fibrin deposition. In addition to the tissue factor pathway, the fibrotic lung environment also activates the intrinsic coagulation cascade. Navaratnam et al. found that levels of factor VIII, a key cofactor in the intrinsic pathway, were significantly elevated in the plasma of IPF patients compared to controls. This increase in factor VIII was associated with worse survival, suggesting a potential link between activation of the intrinsic pathway and disease progression [7]. The fibrotic lung is also characterized by impaired fibrinolysis, which may contribute to the persistence of fibrin clots and the development of a prothrombotic state. Plasminogen activator inhibitor-1 (PAI-1), the primary inhibitor of fibrinolysis, is upregulated in IPF patients’ lungs, correlating with disease severity [21]. The increase in PAI-1 activity may be driven by the profibrotic cytokine TGF-β, creating another point of crosstalk between fibrotic and thrombotic pathways [22]. Beyond the local alterations in the alveolar compartment, IPF is associated with a systemic hypercoagulable state. Plasma levels of D-dimer, a marker of fibrin turnover, are higher in IPF patients and correlate with disease severity [23] and an increased risk of acute exacerbations [8]. This suggests that the local procoagulant environment in the fibrotic lung may “spill over” into the systemic circulation, increasing the risk of thrombotic events in remote vascular beds. The hypercoagulable state in IPF may also be driven by systemic inflammation, another hallmark of the disease. Pro-inflammatory cytokines, such as interleukin-6 (IL-6) and tumor necrosis factor-alpha (TNF-α), are elevated in the plasma of IPF patients [23] and have been shown to activate coagulation and impair fibrinolysis [24,25]. The interplay between inflammation and coagulation may further perpetuate the prothrombotic milieu in IPF.

## 4. Extracellular Vesicles: Linking Fibrosis and Coagulation in Interstitial Lung Diseases

Extracellular vesicles have emerged as crucial mediators in the complex pathophysiology of interstitial lung diseases, particularly IPF [26]. These nano-sized, membrane-bound structures serve as important vehicles for intercellular communication [27] and have been implicated in various aspects of disease progression, including fibrosis and coagulation [28,29]. EVs are lipid bilayer-enclosed structures released by cells into the extracellular space. They can be broadly classified into three main categories based on their biogenesis: exosomes, microvesicles, and apoptotic bodies [30].

### 4.1. Characterization and Biology of EVs in ILDs

EVs are heterogeneous populations of vesicles, primarily categorized into three subpopulations: exosomes (30–150 nm), microvesicles (100–1000 nm), and apoptotic bodies (1–5 μm) based on their size and biogenesis [30]. Exosomes are formed within the endosomal network and released when multivesicular bodies fuse with the plasma membrane [31]. Microvesicles, also known as ectosomes or microparticles, are formed by outward budding and fission of the plasma membrane. Apoptotic bodies are released as blebs from cells undergoing apoptosis [27]. In the context of ILDs, EVs have been detected in various biological fluids, including bronchoalveolar lavage fluid (BALF), blood, and sputum [28,29]. The composition and cargo of these EVs reflect their cellular origin and the pathophysiological state of the lung microenvironment. EVs can carry a diverse range of bioactive molecules, including proteins, lipids, and nucleic acids (mRNA, miRNA, and other non-coding RNAs), which can be transferred to recipient cells and modulate their function. Recent studies have employed advanced techniques such as nanoparticle tracking analysis, flow cytometry, and proteomics to characterize EVs in ILDs [32,33,34,35]. These investigations have revealed that EVs in patients with lung diseases carry a distinct cargo of proteins, lipids, and nucleic acids compared to those from healthy individuals. For example, Njock et al. demonstrated that sputum exosomes from IPF patients exhibit a unique miRNA profile, including elevated levels of miR-142-3p and miR-33a-5p, which are associated with fibrotic pathways [35]. MicroRNAs (miRNAs) are small non-coding RNA molecules that play a crucial role in post-transcriptional regulation of gene expression. The altered miRNA profile in IPF-derived EV suggests their potential involvement in disease pathogenesis and their utility as biomarkers.

### 4.2. EV in Fibrosis

The role of EV in promoting both fibrosis and coagulation in ILDs has been increasingly recognized, positioning them as key players in the complex interplay between these processes [36,37].

EVs contribute to the fibrotic process through multiple mechanisms:

- Pro-fibrotic cargo delivery: EVs from various cell types, including alveolar epithelial cells and activated fibroblasts, have been shown to carry pro-fibrotic mediators such as TGF-β, WNT ligands, and matrix metalloproteinases. These factors can promote fibroblast activation, myofibroblast differentiation, and excessive extracellular matrix deposition [38,39]. TGF-β is a multifunctional cytokine that plays a central role in fibrosis by promoting fibroblast activation, collagen production, and epithelial–mesenchymal transition. WNT ligands are signaling molecules involved in various cellular processes, including fibrosis. Matrix metalloproteinases are enzymes involved in the breakdown of extracellular matrix, but paradoxically, they can also promote fibrosis by activating latent TGF-β and facilitating tissue remodeling.
- miRNA transfer: EV-associated miRNAs, such as miR-21 and miR-29, have been implicated in the regulation of fibrotic pathways. For example, Makiguchi et al. found that serum EV miR-21-5p levels correlate with disease progression in IPF patients [40]. miR-21 is known to promote fibrosis by targeting inhibitors of the TGF-β pathway, while miR-29 is generally considered anti-fibrotic, with its downregulation associated with increased fibrosis [41,42,43].
- Epithelial–mesenchymal transition (EMT) induction: EVs derived from senescent epithelial cells have been shown to induce EMT in neighboring cells, contributing to the expansion of the fibroblast population [38]. EMT is a process by which epithelial cells lose their characteristic properties and acquire mesenchymal features, including increased motility and extracellular matrix production. This process is thought to contribute to the accumulation of fibroblasts and myofibroblasts in fibrotic diseases [44].
- Activation of blood coagulation: The role of EVs in blood coagulation has been extensively investigated. EVs express on the outer membrane phosphatidylserine, a negatively charged phospholipid required for the assembly of the multimolecular complexes that participate in the coagulation cascade. Furthermore, a specific subset of EVs express TF. TF is an integral membrane protein constitutively expressed by tissues outside the blood vessels (and therefore extrinsic to blood). Upon vascular damage, TF becomes exposed to the blood and binds circulating FVII(a), thereby activating FX to FXa and initiating the so-called extrinsic pathway of blood coagulation. Besides this classic pathway, it has been shown that circulating TF, expressed on the surface of EVs, can activate FX [45]. As previously mentioned, FX mRNA is overexpressed in the lungs of human IPF patients compared to normal controls. Furthermore, alveolar cells (A549) exposed to the prooxidant, H_2_O_2_, synthetize FX [46]. We hypothesized that FX activation within the lung is, at least in part, achieved by EV-associated TF. In vitro experiments show that exposure of the same alveolar cells, A549, to H_2_O_2_ increases the release of TF-bearing EVs while not affecting TF synthesis. An observational study in patients with different diseases showed that the bronchoalveolar lavage fluid of interstitial lung disease patients contained significantly higher levels of EV-associated TF activity compared to patients with a different indication of lavage. Furthermore, the activity was significantly correlated to the severity of the interstitial disease, both in terms of lung volumes and diffusion capacity of the lungs [47]. Consistent with a potential role of EV-associated TF, we demonstrated that pirfenidone, a drug that slows down the fibrotic process in idiopathic pulmonary fibrosis, inhibits the expression of TF-bearing EV in A549 cells exposed to H_2_O_2_ via inhibition of p-38 [48].

**Figure 2:**
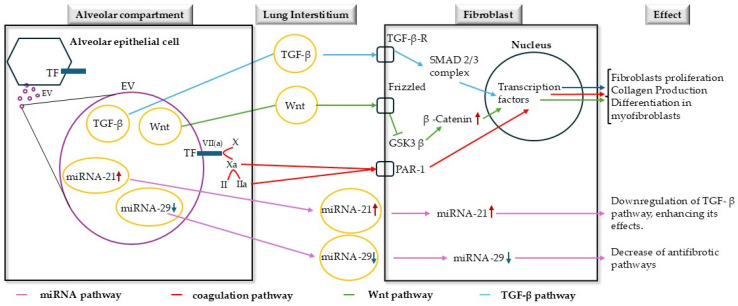
Molecular pathways in pulmonary fibrosis involving extracellular vesicles, coagulation, and fibroblast activation. The figure depicts three key compartments: the alveolar space, lung interstitium, and fibroblast, illustrating how they interact in pulmonary fibrosis. In the alveolar compartment, epithelial cells release extracellular vesicles (EVs) containing multiple bioactive molecules. These include tissue factor (TF), transforming growth factor-β (TGF-β), Wnt, and microRNAs (miRNA-21 and miRNA-29). When released into the interstitium, these molecules trigger distinct but interconnected pathways. The coagulation cascade begins when TF activates factor X to Xa, which then generates thrombin (IIa). Both Xa and IIa activate PAR-1 on fibroblasts. Simultaneously, TGF-β binds to its receptor (TGF-β-R) to activate SMAD 2/3 signaling, while Wnt engages the Frizzled receptor to inhibit GSK3β, leading to β-catenin accumulation. These pathways converge in the fibroblast nucleus, where transcription factors coordinate three main outcomes: fibroblast proliferation, collagen production, and differentiation into myofibroblasts. The process is further regulated by microRNAs, with elevated miRNA-21 enhancing TGF-β signaling and reduced miRNA-29 diminishing antifibrotic responses.

Figure 2 provides a comprehensive overview of the molecular pathways involved in pulmonary fibrosis, focusing on the interplay between EVs, coagulation, and fibroblast activation within distinct lung compartments.

## 5. Therapeutic Implications

The recognition of the pivotal role of thrombosis in IPF pathogenesis and outcomes has spurred interest in targeting thrombotic pathways as a potential therapeutic strategy. However, the optimal approach to thromboprophylaxis in IPF remains uncertain, and several challenges need to be addressed to translate this concept into clinical practice.

### 5.1. Anticoagulation in IPF: Promise and Pitfalls

The role of anticoagulation in idiopathic pulmonary fibrosis has been controversial. Initial interest was driven by observations of activated coagulation in IPF pathogenesis, leading to studies exploring anticoagulation as a therapeutic strategy. The previously mentioned study based on Danish registry data showed that subjects ever treated with anticoagulants had a significantly lower risk of developing idiopathic interstitial pneumonia than patients who were never treated with anticoagulants despite a diagnosis of venous thromboembolism [49]. An open-label trial has randomized IPF patients to receive either prednisolone alone (that represented the standard of care for IPF at that time) or prednisolone plus anticoagulants. There was a significant survival advantage in patients treated with anticoagulants [50]. However, further real-world evidence and clinical trials have yielded concerning results. In a retrospective cohort study, Tomassetti et al. found that IPF patients receiving anticoagulation for cardiovascular indications experienced worse survival (adjusted HR 3.1, 95% CI 1.4–7.0) and shorter time to disease progression compared to non-anticoagulated patients [51]. These findings were corroborated by the ACE-IPF trial, a prospective randomized controlled study that was terminated early due to excess mortality in the warfarin arm (adjusted HR 4.85, 95% CI 1.38–16.99) compared to placebo [52]. It should be noted that at the time this study was performed, prednisolone was no longer the standard of care for this condition. It can be hypothesized that the beneficial effect of anticoagulants in the Kubo study was due to their protective effect against the potential procoagulant activity of prednisolone [53]. While direct oral anticoagulants offer improved safety profiles compared to warfarin in various thrombotic conditions [54], their potential role in IPF remains unexplored. The current evidence recommends against anticoagulation unless indicated for other reasons.

### 5.2. Targeting Specific Coagulation Pathways

The therapeutic approach to pulmonary fibrosis has expanded beyond conventional anticoagulants to focus on specific coagulation pathways. Protease-activated receptors have emerged as key therapeutic targets, with particular attention on PAR-1 and PAR-2. PAR-1, activated by thrombin and factor Xa, plays a central role in promoting fibroblast proliferation and extracellular matrix production [55]. Direct thrombin inhibition in experimental models has demonstrated a reduction in lung collagen accumulation and connective tissue growth factor mRNA levels, supporting the therapeutic potential of targeting this pathway [56]. The TF pathway represents another promising target, with increased expression documented in IPF lungs. TF, working in concert with factor VIIa, activates PAR-2 signaling, which has been shown to stimulate fibroblast proliferation and ECM production [15,55]. While preclinical studies targeting these pathways have shown promise, clinical translation remains challenging [57]. Current evidence suggests that selective targeting of specific coagulation pathways, rather than broad anticoagulation, may provide a more effective therapeutic strategy. However, further clinical trials are needed to establish the safety and efficacy of these targeted approaches in IPF patients.

### 5.3. Profibrinolytic Therapies

The dysregulation of fibrinolysis plays a crucial role in the pathogenesis of idiopathic pulmonary fibrosis, presenting a compelling therapeutic target. The fibrinolytic system, primarily regulated through the interplay between plasminogen activators (uPA and tPA) and their inhibitor PAI-1, maintains a physiological balance between fibrin formation and degradation [58]. In IPF, elevated PAI-1 expression, particularly in alveolar epithelial cells, contributes to reduced fibrinolytic activity and subsequent fibrin accumulation in the pulmonary compartment [59]. This imbalance is further complicated by the dual nature of the plasminogen activation system, as localized increases in plasmin formation in the lung interstitium may paradoxically promote fibrosis through various mechanisms, including activation of matrix metalloproteases and latent growth factors [58]. Recent advances in inhalation therapy have opened new possibilities for targeting this system with improved precision. Inhaled delivery systems, including advanced particle engineering and smart delivery platforms, offer the potential to modulate fibrinolytic activity specifically within the pulmonary compartment while minimizing systemic effects [60]. This approach could overcome the limitations of systemic fibrinolytic therapies, particularly their associated bleeding risks. However, translation of these therapeutic strategies requires careful consideration of the complex spatiotemporal dynamics of the fibrinolytic system in IPF pathogenesis, and robust clinical trials are needed to establish their safety and efficacy.

### 5.4. Predictive Biomarkers and Personalized Medicine

The heterogeneous nature of idiopathic pulmonary fibrosis and its complex pathogenesis necessitates the development of reliable biomarkers for personalized therapeutic approaches. Current research has identified several promising molecular markers that may help stratify patients and predict disease outcomes. These include proteins involved in epithelial cell injury, immune dysregulation, and extracellular matrix remodeling [61,62]. Genetic profiling has emerged as a particularly promising avenue for personalized medicine in IPF. Genome-wide association studies have identified several genetic variants associated with disease susceptibility and mortality [63]. These genetic markers, combined with molecular biomarkers, could potentially create comprehensive predictive models for disease progression and treatment response [62]. The integration of multiple biomarker types—including genetic variants, protein markers, and clinical parameters—may provide a more nuanced understanding of individual disease trajectories. This multi-modal approach could enable more precise patient stratification and guide personalized therapeutic decisions [61,62]. Future research should focus on validating these biomarker panels in large, prospective cohorts to establish their clinical utility in personalizing IPF management.

### 5.5. Combination Therapies and Future Directions

As research in extracellular vesicle therapeutics advances, several critical areas require focused investigation for successful clinical translation. The characterization and standardization of EV isolation, along with detailed analysis of their molecular cargo and biological functions, remain fundamental challenges [64,65]. Key priorities include:

- Optimization of EV Engineering: Development of reproducible methods for EV modification and cargo loading while maintaining their natural biological properties and therapeutic efficacy. This includes establishing standardized protocols for both native and engineered EVs to ensure consistent therapeutic outcomes [65].
- Therapeutic Applications: Further exploration of EVs as both direct therapeutic agents and drug delivery vehicles, particularly in inflammatory and regenerative medicine. This includes investigating their potential to treat various conditions through their inherent immunomodulatory and tissue-regenerative properties [66].
- Clinical Translation: Addressing critical challenges in scaling up EV production, maintaining quality control, and establishing regulatory frameworks. This includes developing standardized methods for EV characterization and potency assessment to meet clinical requirements [65,67].
- Safety and Efficacy Validation: Conducting comprehensive preclinical and clinical studies to evaluate the long-term safety, biodistribution, and therapeutic efficacy of EV-based treatments. This includes investigating potential immunogenic responses and optimizing delivery strategies [66,67].

These advancements will be crucial for establishing EVs as a viable therapeutic platform in clinical practice, particularly in regenerative medicine and inflammatory disease treatment. The complex pathogenesis of IPF suggests that targeting single pathways may be insufficient for optimal disease management. Recent research has revealed interesting mechanistic overlap between antifibrotic and antithrombotic pathways. As mentioned before, we have demonstrated that pirfenidone, a primary antifibrotic therapy for IPF, inhibits the p38-mediated generation of procoagulant microparticles by alveolar epithelial cells. Their findings showed that pirfenidone suppressed H_2_O_2_-induced p38 phosphorylation and subsequent microparticle generation while also reducing microparticle-bound tissue factor activity [48]. This previously unrecognized mechanism suggests pirfenidone may have beneficial effects on both fibrotic and coagulation pathways in IPF. The management of cardiovascular comorbidities is particularly important in IPF patients, as highlighted in comprehensive reviews [68,69,70]. Recent epidemiological data have confirmed a significant burden of venous thromboembolism in IPF patients, underscoring the importance of considering thrombotic risk in disease management [71]. This evolving understanding of the interplay between fibrotic and thrombotic pathways, combined with evidence of comorbidity burden, suggests that comprehensive treatment approaches addressing multiple disease aspects may be necessary for optimal patient outcomes.

## 6. Conclusions

The intricate relationship between pulmonary fibrosis and thrombotic pathology represents a crucial intersection in our understanding of interstitial lung diseases, particularly IPF. This review highlights the bidirectional nature of this relationship, where coagulation factors directly promote fibrotic processes while the fibrotic environment itself activates thrombotic pathways, creating a self-perpetuating cycle that contributes to disease progression. The compelling epidemiological evidence of increased thrombotic risk in IPF patients, coupled with our expanding knowledge of the underlying molecular mechanisms, emphasizes the need for clinicians to maintain a heightened awareness of thrombotic complications in these patients. While early attempts at therapeutic anticoagulation have faced challenges, our growing understanding of specific coagulation pathways and their roles in fibrosis suggests that more targeted approaches may hold promise. Particularly noteworthy is the emerging role of extracellular vesicles as key mediators in this complex pathophysiology. EVs serve not only as biomarkers but also as active participants in both fibrotic and thrombotic processes, representing a novel frontier in our understanding of ILDs. Their ability to carry and transfer bioactive molecules, including procoagulant and profibrotic factors, positions them as crucial players in disease progression and potential therapeutic targets. Looking ahead, several priorities emerge for advancing this field. First, the development of more sophisticated biomarker panels incorporating EV-based markers could improve disease monitoring and risk stratification. Second, the exploration of targeted therapies focusing on specific coagulation pathways rather than broad anticoagulation may yield more favorable risk–benefit profiles. Finally, the therapeutic potential of EV-based approaches, whether through modulation of endogenous EVs or engineered EVs as drug delivery vehicles, represents an exciting frontier in IPF treatment. As we continue to unravel the complex interplay between coagulation and fibrosis, it becomes increasingly clear that successful management of IPF will require integrated approaches that address both processes. The emergence of EVs as both biomarkers and therapeutic targets offers new opportunities for such integrated strategies. Moving forward, careful clinical studies evaluating combination approaches, along with continued investigation of EV biology in ILDs, will be essential for translating these insights into improved patient outcomes.

## Data Availability

Not applicable.
